# Organoids and Spheroids for the Design and the Evaluation of Therapeutic Strategies

**DOI:** 10.3390/pharmaceutics18101199

**Published:** 2026-09-22

**Authors:** Jean-Michel Escoffre, Dévina C. Ung

**Affiliations:** Université de Tours, INSERM, Imaging Brain & Neuropsychiatry iBraiN U1253, 37032 Tours, France; devina.ung@univ-tours.fr

## 1. Introduction

The design and the evaluation of effective therapeutic modalities have long relied on experimental models that only partially reproduce human physiology. Conventional 2D cell cultures (e.g., suspension and monolayer cultures) have provided priceless mechanistic insights but fail to capture the complex cellular interactions, tissue architecture and microenvironment that govern therapeutic responses in-vivo [1]. Animal models remain essential for understanding disease mechanisms and validating therapeutic responses in-vivo. Nevertheless, interspecies differences regularly compromise the prediction of therapeutic efficacy and safety in humans. In addition, increasing ethical concerns and the implementation of the 3Rs principles have further motivated the search for alternative human-relevant experimental models capable of reducing and, whenever possible, replacing animal experimentation [2]. Bridging this translational gap while addressing these ethical challenges has therefore become one of the major objectives in pharmaceutical sciences.

Over the past fifteen years, 3D cellular models—including spheroids, organoids, tumoroids, tissue assembloids, and, more recently, organoid-on-chip systems—have emerged as transformative technologies [1]. These models are progressively redefining how researchers study human physiology, investigate disease mechanisms [1] and design therapeutic strategies [3]. They represent a paradigm change from simplified experimental models toward physiologically relevant human models capable of improving the prediction of efficacy and safety before clinical evaluation.

This advancement can be considered as a succession of technological innovations: (1) The first generation relied mainly on multicellular spheroids, which introduced the concept of 3D cellular architecture and highlighted the significance of cell–cell interactions, diffusion gradients and extracellular matrix organization in determining therapeutic responses [4]; (2) The second generation introduced stem cell-derived organoids capable of self-organization and tissue-specific differentiation [5]. By mimicking critical structural and functional characteristics of native organs, organoids rapidly became powerful platforms for modelling development, disease progression and patient-specific physiology; (3) More recently, a third generation emerged through the convergence of organoid research with bioengineering. Microfluidics, organ-on-chip devices, biomaterials, advanced imaging and computational approaches are progressively transforming organoids into standardized and functional microphysiological platforms [6,7]. These integrated platforms provide unprecedented prospects to improve reproducibility, tissue maturation, and experimental control, thereby enhancing compatibility with drug discovery pipelines.

## 2. An Overview of Published Articles

The present collection of articles perfectly illustrates this incredible progression. Two comprehensive reviews focus on brain organoids and demonstrate how these models are becoming essential tools for neuroscience (Contributions 1 and 5). Beyond reproducing key developmental and pathological characteristics of the human brain, these articles highlight the growing importance of combining cerebral organoids with microfluidic technologies to create more reproducible and physiologically relevant platforms for disease modelling and drug screening. Together, they show the transition from static organoid cultures toward dynamic microphysiological systems capable of better reproducing the complexity of the human brain.

Moreover, a comprehensive review is to tumor organoids (Contribution 2). Patient-derived organoids are quickly transforming precision oncology by preserving the molecular, histological and functional heterogeneity of individual tumors while enabling personalized drug screening. The review also underlines that biomaterial engineering and extracellular matrix design are becoming equally important as cellular biology for realistically mimicking tumor physiology.

Another review addresses 3D liver models, explaining how scaffold-based systems, scaffold-free spheroids, organoids and liver-on-chip technologies are refining the prediction of drug metabolism, efficacy and toxicity (Contribution 3). Because this organ plays a key role in drug pharmacokinetics and in their hepatic clearance, these innovations have direct consequences for decreasing drug failure during preclinical drug design and for building more predictive pharmacological platforms.

Finally, the original research article included in this collection reminds that 3D models are not solely intended for disease modelling (Contribution 4). Indeed, the authors identify novel budesonide analogues capable of maintaining stem cell pluripotency and delaying gastruloid development using gastruloids as developmental models. This work illustrates how increasingly sophisticated 3D systems are becoming valuable tools for mechanistic pharmacology and early drug discovery.

Collectively, these five contributions reveal a significant shift occurring throughout this research field. The question is no longer whether these 3D biological platforms are superior to conventional 2D cell cultures, but how to transform these biologically complex platforms into robust, reproducible and standardized systems suitable for pharmaceutical research, industrial implementation and finally clinical decision-making.

## 3. Perspectives

Despite the remarkable progress achieved over the last decade, several challenges remain. Standardization of culture protocols, reduction of intra- and inter-batch variability, incorporation of vascular, immune and stromal compartments, long-term tissue maturation and compatibility with automation continue to limit widespread adoption. In addition, standardized quality control criteria and clear regulatory frameworks will be essential for their confident integration into drug development pipelines.

Moreover, the next generation of 3D models will emerge through the integration of several rapidly evolving disciplines. Artificial intelligence will progressively assist image-based phenotyping and multiparametric data analysis; synthetic biomaterials will offer better control over tissue microenvironments; patient-derived induced pluripotent stem cells will expand precision biomedical purposes; and interconnected multi-organ platforms will allow to investigate systemic therapeutic responses rather than isolated organs. Together, these innovations will increasingly transform organoids from experimental models into predictive human avatars for drug development.

Ultimately, the future of organoid research should not be measured by their intrinsic complexity, but by their capacity to answer clinically relevant questions with sufficient robustness, reproducibility and scalability to influence therapeutic decision-making. Achieving this objective will require close collaborations between stem cell biologists, bioengineers, material scientists, computational scientists, pharmacologists, clinicians but also regulatory agencies.

We hope that this collection will provide an overview of the current state of the field but also will stimulate future transdisciplinary research aimed at accelerating the translation of 3D models into the next generation of predictive therapeutic platforms.

## Data Availability

No new data were created or analyzed in this study. Data sharing is not applicable to this article.
